# Economic shock and the erosion of COVID-19 precautionary behavior in Canada during the early pandemic

**DOI:** 10.1371/journal.pone.0340685

**Published:** 2026-02-05

**Authors:** Eric Merkley, Peter John Loewen

**Affiliations:** 1 Department of Political Science, University of Toronto, Toronto, Ontario, Canada; 2 College of Arts & Sciences, Cornell University, Ithaca, New York, United States of America; National Taiwan University, TAIWAN

## Abstract

Maintaining voluntary adherence to public health guidelines during a pandemic is fundamentally a collective action problem. We argue that one challenge is the economic costs these behaviors impose on individuals and society. As their costs are revealed to citizens, adherence declines, in part by changing people’s expectations of the behavior of fellow citizens. We leverage the case of the April 2020 Canadian jobs report and use an Unexpected Event during Survey Design (N ~ 4,910) and an Interrupted Time Series to show that the release of this report corresponded with reduced public health adherence, particularly among young panel respondents, and increased aggregate-level mobility. We also use two survey experiments (N ~ 2,500) on national samples of Canadians to show that information about the economic consequences of public health guidelines reduces expectations of adherence by other citizens and by oneself, especially among young respondents. Further, expectations of adherence by others causes expectations of one’s own adherence in the future. The implication is that we need to develop policies that can facilitate pandemic containment without requiring as much costly voluntary behavior on the part of citizens, particularly when the costs of the crisis, and of adherence, are inequitably distributed through society.

## Introduction

The COVID-19 pandemic posed a challenge to governments and citizens unlike any in recent history. Containing its spread, particularly before the roll-out of mass vaccination campaigns, required citizens to adhere to the advice of epidemiologists and public health experts and engage in a wide range of precautionary behaviors, such as maintaining space in public, reducing unnecessary trips outside the home, avoiding workplaces, and limiting one’s in-person social interactions. These actions protected individuals and prevented transmission within the community, but at a cost of serious social and economic disruption. Successful containment hinged to a large degree on individual citizens adhering to public health guidelines voluntarily. Maintaining adherence for an extended and indeterminate amount of time was an enormous public policy challenge, with lessons for how we handle future public health crises.

The dominant approach to the study of these issues has been to focus on how political attitudes shape adherence to public health guidelines. These findings have been valuable but are only one part of the puzzle explaining social, political and public health behaviors. We argue that public health during a pandemic is a public good to which individuals can make a costly contribution by following public health guidelines [1]. These contributions are bound then to decrease when their costs increase, or at least become more apparent, to citizens [2].

We argue that the early COVID-19 pandemic furnished an economic shock to citizens that fundamentally reduced their adherence to public health guidelines. We focus on a series of precautionary behaviors – such as maintaining space in public, reducing unnecessary trips outside the home, and limiting one’s in-person social interactions – often labeled social or physical distancing. The cost of these behaviors was far from trivial. In the case of Canada, two million jobs were reported lost during April 2020, the first full month of the pandemic, as citizens stayed at home and limited social contact to protect themselves and others from infection. Considering this information, citizens may become less inclined to engage in behaviors that contributed to the revealed economic shock. One objective of this paper is to provide evidence that, indeed, adherence to public health guidelines declined because of economic collapse in the early pandemic.

There are several mechanisms that can explain why this might be the case. For one, scholars have shown that the decision to contribute to public goods is a function of its costs and benefits of citizens. They will be less likely to adhere to public health guidelines when the costs become apparent for themselves and for society writ large [1]. For another, economic uncertainty could produce psychological reactance that generates less compliance with public health guidelines [3,4]. Our focus here is on the possibility that economic shocks may erode confidence that other citizens will continue to adhere to public health guidelines in the future. In other words, economic shocks may undermine norm perceptions. Our second objective is thus to shed light on a possible norm-based explanation for how economic shocks erode public health adherence.

Our third objective is to explore sub-group effects in these dynamics. Not everyone benefits equally from adherence to public health guidelines, and COVID-19 is no exception. The risks associated with COVID-19 are far from evenly distributed through the population. Most notably, older citizens are far more at risk of severe complications due to COVID-19 [5]. We thus expect younger individuals to be most affected by information revealing the economic costs of public health guidelines. They have less to gain from maintaining precautions amidst deteriorating economic conditions and eroding expectations that others will do the same.

We provide several pieces of evidence in favor of our theory. First, we test our expectation that economic shocks erode public health adherence by leveraging the case of the April 2020 jobs report in Canada. This official release of employment statistics revealed the size and scope of Canada’s economic collapse at the start of the COVID-19 pandemic – more than 2 million jobs lost and a doubling of the unemployment rate – that far exceeded the forecasts of economists. We use panel-based survey responses with re-contacts occurring before and after the release of the jobs report, along with aggregate-level mobility data to test whether the jobs report reduced adherence. We find consistent evidence that this was indeed the case, especially among younger respondents, though we cannot use these data to shed light on our posited norm-based mechanism.

Second, we present the results of experiments conducted on nationally representative samples. We show that exposure to information about the economic costs of public health guidelines is associated with lower expectations of adherence by other citizens and by oneself, especially among younger respondents. We also provide evidence that exposure to information about descriptive norms around public health adherence causally affects willingness to adhere to public health guidelines.

The central implication of our findings is that costly precautionary behaviors may be difficult to sustain as catastrophic economic costs mount. Government policy needs to acknowledge the possibility that costly, voluntary precautionary behaviors may not be sustainable and consequently accelerate investment in solutions that make society less reliant on such methods, such as testing and tracing, the provision of freely and readily available high-quality masks, improved ventilation, and mass vaccination. Importantly, we argue that these behaviors extend to other forms of mass collective action which require uneven economic sacrifices across generations, income groups, geographies, or other common divisions in democratic societies.

Looking forward, our analysis also reinforces the need of governments to do all in their power to cushion the economic fallout of pandemics and other large-scale societal disruptions with fiscal and monetary policy, especially for those who stand to benefit the least from adherence to public health guidelines and face a disproportionate share of its costs. Pandemics are not without distributional consequences and these citizens are more at risk of abandoning adherence in the future. Additional research should be conducted to examine whether government fiscal policy mitigates the effects we see here. There is reason to expect so [see 4].

### Physical distancing as public goods co-production

Containment of the COVID-19 pandemic required citizens to engage in widespread social or physical distancing on the advice of epidemiologists and other health experts. Understanding what groups of citizens are likely to take heed of expert advice is thus vitally important. Many scholars see the central challenge as dealing with individual-level predispositions often at odds with expert advice. For example, trust in experts appears to be centrally important cross-nationally [6,7], while partisanship played an outsized role in shaping the public’s response to the pandemic in the United States [8,9].

This approach is valuable, but its emphasis on understanding *attitudes* has some important limitations when applied to understanding the *behavior* of citizens. Citizens tend to form attitudes in line with their social identities because motivations toward direction can dominate accuracy in politics [10]. They do not face costs for holding inaccurate beliefs, while they gain social-psychological benefits for supporting their social groups. Individual decisions to engage in social or physical distancing, however, have clear costs and benefits for themselves that also partially spill over to other citizens. They are behaviors with consequences.

Public health provision, such as the sanitation, clean air and water, and widespread vaccination, has some of the characteristics of a public good. It is non-excludable and mostly non-rivalrous [11]. However, provision of this good can be partially characterized by its co-production by governments and citizens [12]. In a democracy some level of public health production – likely a high level – is going to be dependent on the voluntary actions of individual citizens and their willingness to adhere to health directives.

Consequently, the level of public health co-provision is going to be partially determined by the costs and benefits of participation for citizens [1,2]. This is a problem because physical distancing entails enormous short and long-term economic and social costs on society [13]. As citizens face the high economic costs of physical distancing, we should expect reduced adherence [1,2]. This pattern is not unique to pandemics. For instance, scholars have shown that the willingness of citizens to adopt pro-environmental attitudes and behaviors is shaped by economic conditions and the costs and benefits of policies [14–16].

During the COVID-19 pandemic, the economic costs of voluntary physical distancing and government lockdowns were strongly brought to the forefront of political discussion with monthly releases of unemployment data. We focus on the April jobs report in Canada, released on May 8, 2020, that revealed the unemployment rate had increased to over 13% in April with 2 million jobs lost – by far the worst single monthly jobs report in Canadian history. Existing work has examined whether individual-level economic insecurity is associated with less public health adherence [3,4]. Here we exploit a discontinuity in the information environment surrounding the economic costs of the pandemic by using individual-level survey data in which respondents self-reported their public health adherence and Google mobility behavioral data through April and May. Our expectation is that the April jobs report produced a reduction in self-reported adherence and an increase in mobility.

H1: The release of the April jobs report (A) and exposure to negative economic information (B) is associated with less public health adherence.

Understanding adherence to public health guidelines must account for the fact that some groups of citizens receive comparatively less benefit from adherence than others. Perhaps most notably, older citizens are far more at risk of serious complications from COVID-19 than those who are younger [5]. The economic fallout of the pandemic is also especially devastating for younger citizens, dramatically reducing income and opportunity in prime earning years, particularly since younger citizens make up a disproportionate share of the devastated service sector [17]. We expect the April jobs report to have had stronger effects on younger respondents.

H2: The negative effect of the April jobs (A) report and negative economic information (B) on public health adherence is stronger among younger respondents.

### Social norms and public health adherence

We will show that the April jobs report reduced public health adherence, particularly among the young. Certainly, some of these effects are because people became less inclined to take actions that were revealed to be costly for themselves and society. But we believe that some of this effect operates through changes in people’s expectations of the behavior of others. People’s perceptions about what others will do are also important in shaping how they behave in a wide variety of contexts [18]. That is, citizens have certain subjective perceptions of the norms that are prevalent in society. They will engage in what they perceive as norm compliant behavior to avoid social sanction, to feel a sense of belonging in their community [19], or out of a need to be ‘liked’ by those in their social group [20]. Norm following can also arise as an automatic response to cues in their social environment [18].

It is not surprising, then, that changing norm perceptions are often touted as a vehicle to affect pro-social behavioral change [21]. Most efforts revolve around changing people’s descriptive and injunctive norm perceptions, where the former involves conveying descriptive information about how many people in some relevant referent group engage in pro-social behaviors, while the latter uses information about what behaviors group members think others *should* engage in [21,22]. Descriptive and injunctive norms often go hand-in-hand, but this is not necessarily the case. For instance, providing injunctive norms alongside descriptive information that suggests widespread non-adherence may produce a backlash effect that undermines the norm [23].

A large literature in social psychology has examined the effects of norm perceptions and social influence on a diverse array of attitudes and behaviors, many of which reflect collective action or public goods provision. Most crucially, norm perceptions have been found to associate with a variety of health behaviors like exercise [24], reduced binge drinking [25], cancer screening [26], and sunblock usage [27]. Descriptive or injunctive norms can influence behavior when people observe the behavior of individuals [28], groups [29], and institutions [21]. All these dynamics were likely operating during the COVID-19 pandemic, as adherence to COVID-19 guidelines involved very public acts that others could observe, public opinion polling communicated information about the public’s adherence to public health guidelines, and health officials and government institutions reinforced the need for citizens to distance themselves from others in their communications.

It is not surprising, then, that existing research shows norms are important in explaining why citizens engaged in a variety of protective behaviors throughout the COVID-19 pandemic. Work has found observational correlations between norm perceptions and self-reported precautionary behavior [6,30–33, but see 34]. Providing information on norms around COVID-19 vaccination may increase vaccination intention [35, but see 36]. Conversely, showing examples of noncompliant behaviors related to physical distancing and vaccination may increase resistance to public health guidelines [37].

Much less work, however, has examined whether changes in the social, political, or economic context can shape people’s COVID-19 norm perceptions. We contend that information on the economic costs of public health guidelines like physical distancing reduced expectations that other citizens would maintain their adherence with these guidelines for the long-term. This happens because people recognize that others will be reticent to adopt attitudes and behaviors that are costly to themselves or society, all else being equal.

H3: Information on the economic cost of public health guidelines reduces expectations of public health adherence by other citizens.

We are not able to shed light on this norm-based mechanism with our observational data, so we turn to a survey experiment that randomly assigns a conservative projection of the economic fallout of public health guidelines to examine its effect on people’s beliefs that other citizens will adhere to public health guidelines in the future. In this experiment, we are also able to examine hypotheses analogous to H1 and H2 (labelled H1B and H2B), because we believe that this information should ultimately undermine one’s own expected adherence to public health guidelines, especially among younger respondents.

There are only a few experimental studies that exposed respondents to economic information about the pandemic. Normand et al. (2022) found that students primed to think about economic insecurity reduced expectations of their public health adherence [3]. Instead, we focus our treatment on information provision (i.e., an economic forecast) on a large, nationally representative sample, and measure expectations of *others’* adherence post-treatment, as well. Dylong and Koenings (2023) manipulate the framing of otherwise equivalent economic information to examine effects on policy support [38], but they were not interested in explaining public health adherence or expectations of others’ behavior.

Our theory implies mediation: that information exposure reduces expected adherence *through* changes in the expectations of others’ behavior. While we do not evaluate mediation directly in this manuscript [39], we do test the grounding assumption that changing expectations of others’ adherence with public health guidelines changes one’s own expected adherence. We conducted a second experiment where we randomly assigned respondents polling information on the public’s willingness to engage in a variety of precautionary measures to examine its effect on one’s own expectations of adherence.

H4: Changing expectations of the public health adherence of other citizens affects expectations of one’s own adherence.

## Methods – Unexpected event during survey design

Economists and public policy experts expected the COVID-19 pandemic to have negative consequences for the national economy. Government lockdowns shuttered most non-essential businesses, while individual citizens limited their social and economic activity to “flatten the curve.” However, typical indicators of economic performance are ill-equipped to shed light on such a rapidly unfolding situation. Canadians were in the dark on the full scope of the economic damage until Statistics Canada released the results of its April labor force survey on May 8, 2020. This was the first complete picture of the economic consequences of the early COVID-19 pandemic.

The report revealed that 2 million jobs were lost over the course of April – the highest monthly figure on record – with young people bearing the brunt of the shock. The unemployment rate in Canada rose to 13%, which was likely an underestimate since recently employed people dropped out of the labor force because of the pandemic and are not included in the calculation of that statistic. Statistics Canada estimated the unemployment rate was closer to 18% after adjusting for this fact. This was the worst jobs report in Canadian history.

The April jobs report provided information to the public about the economic costs of controlling the COVID-19 pandemic. In the Supplementary Materials (S5 and S6 Figs in S1 File) we illustrate how economic news changed in the aftermath of the report and show that the report led citizens to massively update their economic perceptions. We expect that this shock reduced public health adherence (H1A), especially among young people (H2A). We test the effects of the April jobs report on public health adherence first by using an Unexpected Event during Survey Design (UESD). This is an approach that exploits the occurrence of an event during the fielding of a survey to examine its causal effects on an outcome variable. A USED effectively uses the timing of the survey to create an instrument for exposure to the event [40] – in this case the April jobs report.

We test this by making use of surveys conducted by the Media Ecosystem Observatory (MEO). The MEO conducted seven surveys of approximately 2,500 adult Canadian citizens each between April 2 and May 27, 2020 using the sample provider Dynata. Quotas were set on gender, age, language, and region, and we further weight our data within region by age and gender. The MEO surveys, in addition to the experiments provided below, were approved by the University of Toronto Social Sciences, Humanities and Education Research Ethics Board (Protocol #00038251). All participants gave their written, voluntary, informed consent upon entering the surveys used in this manuscript and were given the ability to withdraw their data upon request without penalty. No minors were contacted for these surveys. The fielding dates and sample size of each wave can be found in the Supplementary Materials (S6 Table in S1 File).

In each wave of the survey, we ask respondents whether or not they have taken a variety of actions over the previous week in response to the pandemic. We use three of these items that most closely reflect the public health guidelines provided by health experts (i.e., avoided in-person contact; kept a distance of two meters; avoided domestic travel) to construct an additive index of our central outcome. Another item – avoiding bars, restaurants and crowded places – was omitted from this index because it is likely to be strongly shaped by government lockdown policy at the time these surveys were fielded and thus less reflective of individual choices. Respondents would not have had the opportunity to go to bars or restaurants even if they wanted to do so until late May.

UESD relies on two central assumptions. The first is excludability: that the timing of the survey does not affect the outcome in any way aside from the event of interest [40]. The second assumption is ignorability: when the treatment (i.e., exposure to the jobs report) is independent of potential outcomes [40]. Given that the treatment is not randomly assigned in the UESD framework, anything that affects the probability with which respondents are assigned treatment can pose a threat to causal inference (e.g., covariate imbalance, differential attrition, etc.).

An ideal event for a USED is one that is entirely unexpected. The April jobs report does not fully meet this criterion since it is a regularly scheduled monthly release. In this case, however, the content of this report was indeed unexpected. As noted, the scale of jobs loss revealed by the report exceeded the worst forecasts by professional economists by a substantial margin (used to design the treatment for Experiment 1 below), precipitating a torrent of negative economic news (see the Supplementary Materials). That it is regularly rescheduled also mitigates worries that the report’s release could be endogenous to the political and economic environment.

We take several steps to lessen – though not entirely eliminate – threats to causal inference posed by excludability and ignorability. First, we exploit the fact that around half of respondents in the first three surveys were re-contacted. Approximately half of waves 4–7 were re-contacts of waves 1–3 (N ~ 3681). As a result, some of these re-contacts occurred before the jobs report and some occurred after its release during the fielding of wave 5. We can thus examine whether those re-contacted after the report exhibited sharper *within-respondent* decreases in precautionary behavior compared to those re-contacted before the report release. Helpfully, there is no significant trend in within-respondent change in precautionary behavior in the control period before the release of the report, which helps us meet the excludability assumption. Recontacted respondents have similar levels of political interest and education. They are also demographically quite similar, though we had a marginally harder time recontacting young people (see S7 Table in S1 File).

Second, balance tests reveal that age and education are systematically correlated with whether respondents were surveyed in the post-jobs report period (see S8 Table in S1 File). We use entropy balancing to reduce covariate imbalance in our estimation of treatment effects to meet a more relaxed conditional ignorability assumption: independence from potential outcomes conditional on covariates.

Our principal model is as follows:


$${\Delta Y}_{i}=\;\alpha +\;\beta_{\text{1}}X_{i}+\;\beta_{\text{2}}Z_{i}+\;\varepsilon$$

Where ${\Delta Y}_{i}$ is the within-respondent change in precautionary behaviors between the contact and re-contact periods, $\beta_{\text{1}}$ is the estimated effect of the jobs report release (adjusted for covariate imbalance), and $\beta_{\text{2}}$ is the estimated effect of provincial reopening, which we treat as the date the province of the respondent reopened retail stores. we include a lag such that the binary variable for the jobs report release takes on a value of 1 for May 9 and onward. The reasoning is that respondents are being asked to retrospectively evaluate their public health precautions over the previous week. We use heteroskedastic-robust standard errors in all specifications.

Third, we repeat this estimation with two narrower bandwidths. The first, which we call *Narrow 1* removes respondents re-contacted in the final wave, fielded much later, from May 21–27. The second, which we call *Narrow 2*, keeps only respondents who were re-contacted in the waves immediately before and after the report release, fielded between May 1 and 12. These narrower bandwidths allow us more confidence in ruling out simultaneous events and pre-existing trends – threats to excludability. Notably, the *Narrow 2* specification occurs in a period entirely before the provinces moved to reopen their economies.

We include a series of falsification and placebo tests to assess possible violations of excludability. A useful falsification test uses alternative outcome variables that are affected by simultaneous events and also might influence the outcome but are themselves unlikely to be influenced by the treatment [40]. We use two outcome variables which we expect are not influenced by the jobs report. *Falsify 1* uses a COVID-19 risk perceptions additive index based on two questions asking respondents to rate how serious of a threat COVID-19 was to themselves personally and to other Canadians (response options: very serious, somewhat serious, not very serious, not at all serious). We see no reason the jobs report would influence risk perceptions. At the same time, declining COVID-19 cases, government policy reopening the economy, and warming temperatures through this period (thereby facilitating outdoor activities) could influence both risk perceptions and precautionary behavior. *Falsify 2* uses an additive index of respondents engaging in three online or workplace-related behaviors in the past week: 1) worked from home; 2) switched to virtual meetings; 3) switched to online shopping. The jobs report release should not have any effect on these behaviors, but provincial reopening policy may influence all three.

Placebo tests serve a similar purpose by ruling out pre-existing trends. This can be done by creating a pseudo-treatment at some point to the left of the cutoff (i.e., the jobs report release date). The best approach is to use the median time point in the control period to maximize statistical power for the test. So, *Placebo* uses our primary outcome measure but creates a placebo treatment variable at the approximate median of the pre-jobs report re-contact period (May 2).

After establishing the effect of the jobs report release on precautionary behavior, we examine whether treatment effects are heterogeneous by age. We estimate a pair of models using our more conservative *Narrow 2* bandwidth that interacts either a continuous measure of respondent age (in years) or a categorical version of the variable (aged 18–34, 35–54, 55 and older) with a binary variable indicating the respondent was re-contacted in the post-jobs report period. We control for interactions of the report release with anti-intellectualism, ideology, news exposure, education, urban/rural residence. Again, the results do not depend on the inclusion of the controls (see Supplementary Materials, S9 Table in S1 File).

## Results – Unexpected event during survey design

We display the results from our non-interactive models in Table 1. In our base model, we find that being re-contacted in the period after the release of the jobs report is associated with an additional 0.05 point reduction in self-reported public health adherence (p < .001). This amounts to a 0.18 standard deviation decrease on this measure. We can further illustrate the effect size by unpacking how many people reduced their precautionary behaviour in the pre- and post-jobs report period. Almost 19% of people recontacted before the jobs report reported less precautionary behaviour. 83% of these respondents took one fewer action in the recontact survey. After the jobs report, the share of respondents reducing their physical distancing increased by 8.1 percentage points, or around 42%. The share of people reducing their distancing by only one action dropped to 76%, meaning that a modestly higher share of respondents reduced their behaviours by two or more on the index.

**Table 1:** UESD OLS regression estimates.

	Base	Narrow 1	Narrow 2	Falsify 1	Falsify 2	Placebo
DV =	Precautionary behavior	Precautionary behavior	Precautionary behavior	Risk	Workplace behavior	Precautionary behavior
Jobs Report	−0.055***	−0.043**	−0.038**	−0.005	−0.017	
	(0.016)	(0.018)	(0.016)	(0.009)	(0.014)	
Pseudo-intervention						−0.008
						(0.013)
Reopening	−0.023	0.023		−0.031***	−0.001	−0.061***
	(0.018)	(0.024)		(0.010)	(0.016)	(0.012)
Constant	−0.016**	−0.022***	−0.017*	−0.039***	−0.008	−0.018*
R^2^	0.011	0.004	0.004	0.008	0.001	0.010
N	3681	2788	1890	3681	3681	3679

Note: Robust standard errors in parentheses. * p < 0.1, ** p < 0.05, *** p < 0.01. ‘Narrow 1’ model drops respondents re-contacted in the final wave, fielded from May 21–27, while ‘Narrow 2’ preserves only respondents who were re-contacted in wave 5, fielded from May 1–5 immediately before the report release, and those who were re-contacted in wave 6, surveyed from May 8–12. ‘Falsify 1’ uses COVID-19 risk perceptions as the outcome and ‘Falsify 2’ uses an index online and workplace-related behavior. ‘Placebo’ uses a pseudo-treatment date of May 1. Reopening omitted from Narrow 2 since bandwidth excludes reopening dates.

Restricting the bandwidth to remove respondents contacted in late May (*Narrow 1)* reduces the estimated effect to 0.04 (p = .016) or approximately 0.14 standard deviations, while focusing on the re-contact waves immediately preceding and following the jobs report (*Narrow 2)* results in a nearly identical point estimate of 0.04 (p = .017).

Table 1 shows that the jobs report release is not significantly associated with changes in COVID-19 risk perceptions (−0.00, p = .564), nor online or workplace-related behaviors (−0.02, p = .246), which we would expect if time varying confounds like pandemic-related conditions and government policy biased our estimates. We also find no significant association between our placebo treatment date and our outcome, which helps us further rule out confounds from pre-existing trends. These results strongly support H1A.

**Fig 1:**
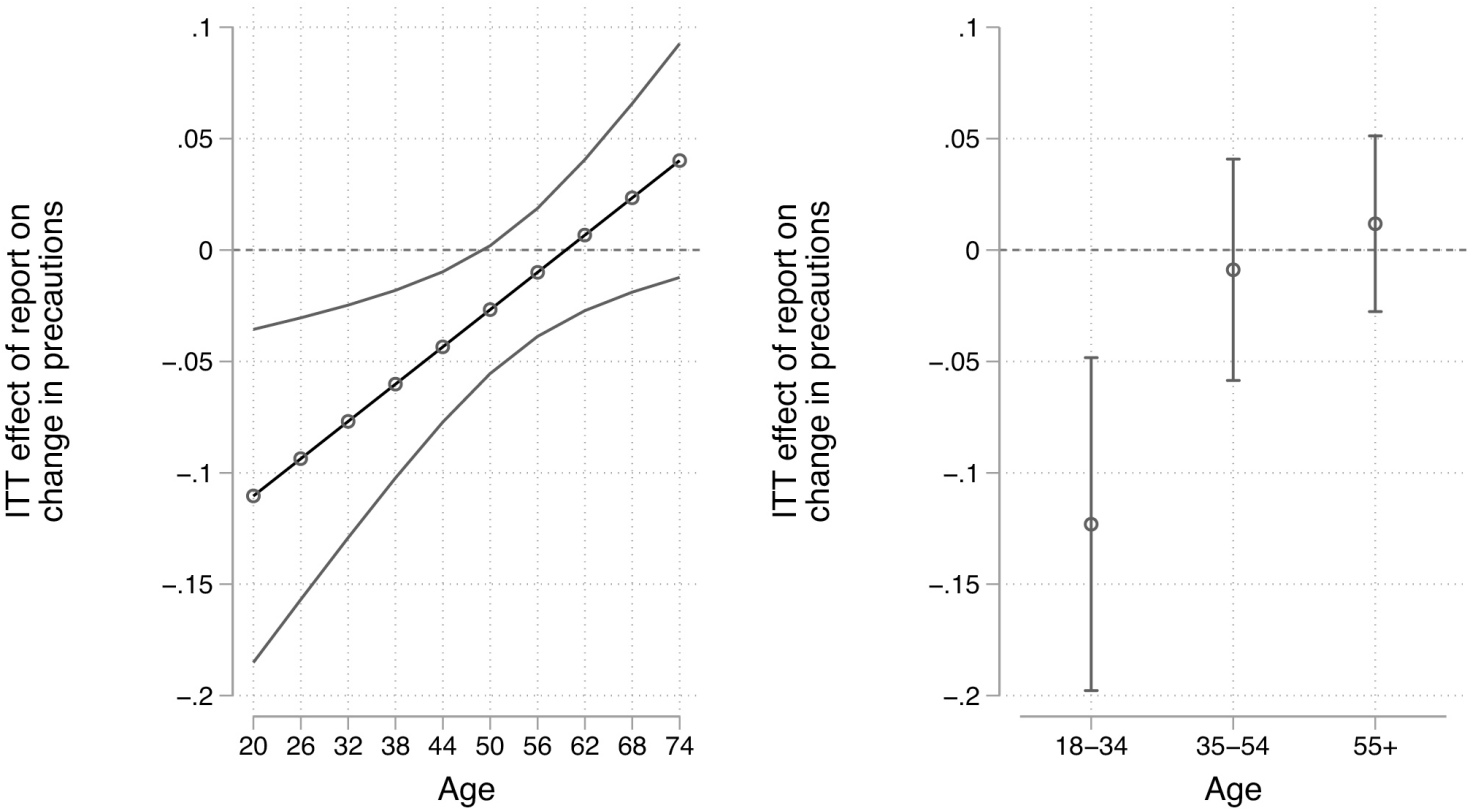
Intent-to-Treat effects of the job report release on changes in public health adherence across age group using continuous (left panel) and categorical measurement (right panel). Note: 95% confidence intervals. The full estimates can be found in S9 Table in S1 File.

We also find evidence of heterogeneous effects across the reported age of our respondents (H2A). Respondents aged 26 years re-contacted after the release of the jobs report are expected to reduce their reported public health adherence by 0.09 points more than those re-contacted beforehand. This effect weakens until it becomes statistically insignificant for those 50 years and older. The interaction term is statistically significant (p = .009). This result is presented graphically in the left panel of Fig 1. The estimates can be found in the Supplementary Materials.

It is possible there is non-linearity in these heterogeneous effects, so we present an additional estimation treating age categorically. Those between the ages of 18 and 34 are most affected by the jobs report release, controlling for other factors. They are expected to reduce their precautionary behavior by 0.12 points (p < .001), which is approximately 0.40 standard deviations. We see no significant effects for those between 35 and 54 (−0.01, p = .726) and those over the age of 55 (0.01, p < .558). The differences between the latter two age groups and those between the ages of 18 and 34 are both significant (p = .012 and p = .002). We find strong support for H2A.

What is driving the age effect? Part of the story is that younger citizens faced steeper economic consequences from pandemic restrictions. And we do see some evidence as well that the effect of the job report was strongest among those who thought their job was at least at some risk due to the pandemic (−0.09, p = .001) compared to those who believed there was no risk to their job (0.00, p = .816). The interaction is significant (p = .003). These results are displayed in S7 Fig in the Supplementary Materials in S1 File.

## Methods – Interrupted time series design

The above analysis has two key limitations. First, it hinges on the reliability of self-reported survey measures of public health adherence during the pandemic. There is considerable ambiguity over how to measure such adherence in a valid and reliable fashion in the social sciences. One potential threat to these measures is social desirability bias. Some work has shown that estimates may suffer upward bias due to social desirability [41], though the gravity of this problem has been contested [42], while others find that these measures nonetheless match behavior reasonably well [43].

Second, our survey design was not one of a rolling cross-section with representative samples within each day, so we do not have continuous daily samples that would allow us to effectively rule out time varying confounders. It is possible there are other changes happening around the time of the jobs report that produce less public health adherence – especially among young people. We alleviate some of this concern by showing effects that are very close to the release of the jobs report and with the results of our falsification and placebo tests, but we cannot rule out this problem entirely.

Consequently, we supplement our individual-level findings with an interrupted time series analysis of Google mobility data from April 1-May 29 in Canada. We cannot examine heterogeneous effects using these data and the level of granularity in the data does not allow us to do fine-grained spatial analysis. We can, however, evaluate whether there is a discontinuity in the mobility series corresponding to the release of the April jobs report after accounting for time-varying confounders.

We create an index of two Google mobility series that we expect are more likely to be a function of voluntary adherence with public health guidelines: retail/recreation, and groceries/pharmacy. We also construct an index of two series that are less likely to reflect voluntary adherence with public health recommendations, but rather occupation and employment patterns: residential (reverse-scaled) and workplace mobility. A principal components analysis shows that these measures load together on two distinct factors as expected (retail (0.24, 0.95), grocery (0.00, 0.98), workplace (0.97, 0.16), residential (0.98, 0.07)). We omit the category of parks because through this period evidence gradually emerged that outdoor transmission of the novel coronavirus was rare, though public policy was slow to catch on. Our results do not hinge on whether this mobility category is or is not included in our outcome measure.

Both of these measures are stationary according to Dickey-Fuller tests. Out of these measures we partial out the effects of the day of the week and three holidays that occur through this period – Good Friday (April 10), Easter (April 12), and Victoria Day (May 18) – by regressing our mobility series on these binary variables and extracting the residuals. We standardize these indices for ease of interpretation.

We use interrupted time series models to estimate the effect of the April jobs report on mobility patterns. These models are increasingly used to examine the effect of public health interventions on behavior and health outcomes when randomized control trials are not possible. They allow scholars to identify immediate (i.e., a level change) effects of an intervention and changes in the underlying trend in an outcome induced by an intervention (i.e., a slope change) [44].

The core model is as follows:


$$Y_{t}=\;\alpha +\;\beta_{\text{1}}T+\beta_{\text{2}}X_{t}\;+\beta_{\text{3}}{TX}_{t}+\;\varepsilon$$

Where $Y_{t}$ is our mobility outcome variable and $\beta_{\text{1}}$ is interpreted as the trend in the outcome before the jobs report. $\beta_{\text{2}}$ represents the immediate effect of the jobs report (i.e., level change) and $\beta_{\text{3}}$ represents the change in the trend post-jobs report (i.e., slope change). We strongly expect to observe a level change in mobility induced by the report. We might also expect a slope change as people are gradually exposed to the information in the news media.

Unbiased estimates of the immediate and long-term effects of the jobs report hinge on the absence of other time varying confounders. There are three that are of particular interest. First, and most importantly, provincial governments began reopening their economies through this period. We treat provincial-level reopening as a second intervention that we incorporate into our interrupted time series modeling. We use May 18 for the start date of this intervention because it was the date when Ontario and British Columbia reopened their retail stores.

We also control for current and lagged average daily temperature and lagged daily confirmed COVID-19 cases. The weather improved and new confirmed cases fell during this period, so we want to account for these influences on mobility that may be correlated with the release of the report. Weather data was taken from the Government of Canada’s historical data weather series for their station at Pearson Airport in Toronto. Daily confirmed COVID-19 cases was downloaded from the Government of Canada’s COVID-19 dashboard.

We estimate two principal models. The first includes the jobs report and the retail reopening interventions. The second adds controls for temperature and COVID-19 cases. We also estimate two “placebo” models. The first uses our workplace and residential mobility series instead where we expect to find no effect of the jobs report. The second uses a pseudo-intervention start date at the median of the pre-intervention period [following 44]. Using the median maximizes statistical power to detect a placebo effect. We use Newey-West standard errors to account for serial correlation up to highest lag as detected by Cumby-Huizinga tests for serial correlation.

Google also offers province-level mobility series. Data is extremely sparse for Prince Edward Island, so this province is dropped from these analyses. Although the national-level jobs report is not expected to differentially effect citizens in various provinces, we can more effectively control for province-level temperature, daily COVID-19 cases, and reopening policies. We use the opening date for retail for province-level reopening in these models. Note that Newfoundland and Nova Scotia did not reopen retail through this period, so they are scored ‘0’.We use data from the weather station at the airport of the largest city in each province. We use a generalized linear model to estimate our parameters of interest, with standard errors adjusted to account for serial correlation up to highest lag as detected by Cumby-Huizinga tests.

## Results – Interrupted time series design

Table 2 presents the estimates for our interrupted time series analysis using national-level aggregate mobility data. There is strong evidence for a change in the level of mobility induced by the job report, supporting H1A. Model 1 shows that the jobs report had a 0.59 standard deviation effect (p = .01) on the level of consumer-related mobility (i.e., retail/recreation, groceries/pharmacy). Provincial reopening also had an important effect on this type of mobility – increasing mobility by approximately one standard deviation (p = .002). Model 2 introduces the controls for temperature and confirmed daily COVID-19 cases. The effects of these variables are not statistically significant and the estimate for the jobs report remains mostly unchanged.

**Table 2:** National Mobility Interrupted Time Series Estimates.

	Principal Models		Placebos	
DV =	Mobility	Mobility	Workplace mobility	Mobility
Time	0.03***	0.02**	0.04***	0.05*
	(0.01)	(0.01)	(0.01)	(0.03)
Jobs Report	0.59***	0.57*	0.16	
	(0.22)	(0.34)	(0.28)	
Job Report * Time	−0.02	−0.03	−0.02	
	(0.04)	(0.04)	(0.05)	
Pseudo-intervention				−0.65*
				(0.33)
Pseudo-intervention * Time				−0.00
				(0.04)
Reopening	1.03***	1.01***	0.53	0.79***
	(0.32)	(0.20)	(0.41)	(0.22)
Reopening * Time	−0.00	−0.03	0.03	−0.08**
	(0.05)	(0.04)	(0.06)	(0.03)
Temperature _t_		−0.02	0.01	−0.02
		(0.02)	(0.02)	(0.02)
Temperature _t-1_		0.03	−0.01	0.02
		(0.03)	(0.02)	(0.02)
Daily Cases _t-1_		−0.00	−0.00*	−0.00*
		(0.00)	(0.00)	(0.00)
Constant	−1.12***	−0.73	−0.70	−0.41
Lags	0	3	0	0
N	59	58	58	58

Note: Newey-west standard errors at the specified lag in parentheses. * p < 0.1, ** p < 0.05, *** p < 0.01.

**Fig 2:**
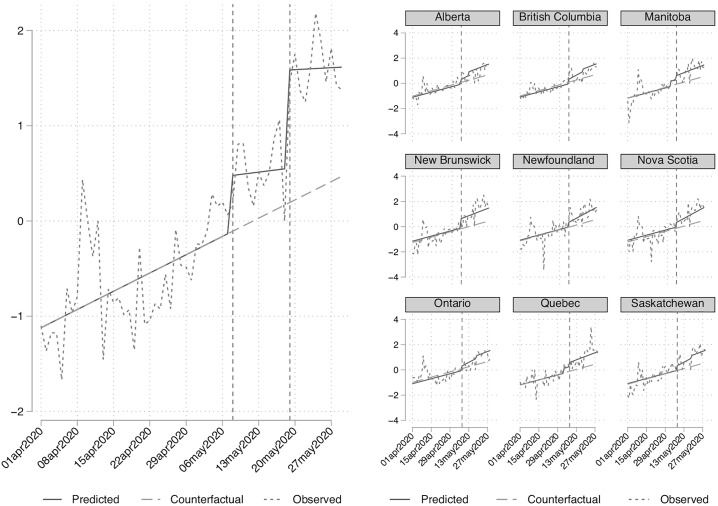
Predicted mobility compared to counterfactual trend for national estimates (left) and provincial estimates (right). Vertical dashed lined in left panel denote days for the jobs report release and then reopening. Vertical dashed line in right panel denotes day of jobs report release. Predictions from left and right panels derived from estimates in Tables 2 and 3, respectively. The series displayed are adjusted for holiday and day-of-week effects. A comparison of the raw and adjusted series can be found in S8 Figure in the Supplementary Materials in S1 File.

The left panel of Fig 2 plots the predicted values of mobility from Model 1, the observed values, and the counterfactual trend if neither the jobs report nor provincial reopening occurred. There is a clear discontinuity at the time of the jobs report release. Confidence in our inference is heightened by our placebo estimates in Models 3 and 4. We do not observe statistically significant effects in the expected direction on workplace-related mobility, nor for our pseudo-intervention date at the median between the start of the study period and the release of the jobs report.

**Table 3:** Provincial Mobility Interrupted Time Series Estimates.

	Principal Models		Placebos	
DV =	Mobility	Mobility	Workplace mobility	Mobility
Time	0.03***	0.03***	0.04***	0.05***
	(0.00)	(0.01)	(0.00)	(0.01)
Jobs Report	0.37***	0.36***	−0.04	
	(0.11)	(0.10)	(0.06)	
Job Report * Time	0.03**	0.02*	0.02**	
	(0.01)	(0.01)	(0.01)	
Reopening	0.97**	0.94*	0.35	0.65
	(0.49)	(0.51)	(0.43)	(0.40)
Reopening * Time	−0.02	−0.02	−0.00	−0.01
	(0.01)	(0.01)	(0.01)	(0.01)
Pseudo-intervention				−0.72***
				(0.12)
Pseudo-intervention * Time				0.00
				(0.01)
Temperature _t_		0.02***	0.01**	0.02***
		(0.01)	(0.01)	(0.01)
Temperature _t-1_		−0.01*	−0.01*	−0.01
		(0.01)	(0.00)	(0.01)
Daily Cases _t-1_		−0.00**	−0.00***	−0.00***
		(0.00)	(0.00)	(0.00)
Constant	−1.80***	−1.55***	−1.13***	−1.20***
Lags	10	10	11	13
Fixed effects	Yes	Yes	Yes	Yes
Groups	9	9	9	9
T	59	58	58	58
N	531	522	522	522

Note: Robust standard errors in parentheses. Autocorrelation structure set at specified lag * p < 0.1, ** p < 0.05, *** p < 0.01.

Table 3 presents the provincial mobility estimates. Model 1 shows that the jobs report increased the level of mobility by about 0.37 standard deviations (p = .001), again supporting H1A. It also affected the slope of mobility moving forward (p = .019), roughly doubling the daily trend to 0.06 standard deviations (p < .001). Provincial reopening policy also had an effect, increasing the level of mobility by 0.97 standard deviations (p = .045), but with no statistically significant slope change (p = .166). Model 2 includes controls for temperature and confirmed daily cases. We see no meaningful change in the estimates. Both temperature and confirmed cases appear to be related to mobility in their own right. A short-term increase in temperature of 3 degrees (approx. one standard deviation) is expected to increase mobility by 0.06 standard deviations. An increase in the previous day’s cases by 350 (approx. one standard deviation) is expected to reduce mobility by 0.04 standard deviations.

The right panel of Fig 2 plots the predicted values of mobility from Model 1, the observed values, and the counterfactual trend if neither the jobs report nor reopening occurred for each province. Again, there is a clear discontinuity at the time of the jobs report release. Our placebo models (3 and 4) also show mixed evidence of the jobs report on workplace mobility. There is no evidence of a level change (p = .566), though the slope does appear to modestly increase at this period (p = .014). The level change for the pseudo-intervention date is significant (p < .001), but it is signed in the wrong direction, while the slope did not change (p = .952). All told, it does appear that there is an important discontinuity in mobility related to retail and recreation at the time of the jobs report release that cannot be accounted for by provincial reopening or other time varying confounds.

## Methods – Survey experiments

We conducted a pair of survey experiments to provide causal identification of a link between information about economic costs and public health adherence and to shed light on a possible norm-based mechanism producing these effects. These experiments were embedded in the MEO surveys used for the UESD design presented above.

### Experiment 1

We surveyed 2,499 Canadian citizens 18 years or older from April 2–6, 2020 using sample provided by Dynata in an identical fashion to the MEO surveys from above. Complete sample characteristics can be found in S1 Table of the Supplementary Materials in S1 File. We randomly assigned our respondents information on the forecasted economic consequences of public health measures to examine its effect on expectations that others will comply with these guidelines and whether they would do the same. Our expectation is that exposure to this information will lower expectations of adherence across the board.

We believed that some of the treatment effect is mediated by changes in expectations of 1) other people’s compliance and 2) government policy. Consequently, we crossed our economic cost randomization with polling information about public health compliance, and government policy in order to manipulate these mediators. We provide a descriptive path model and the results of these experimental analyses in the Supplementary Materials. The results provide some suggestive evidence of mediation, but we were not successful in exogenously manipulating our mediators to allow for causal identification.

In this experiment, all respondents received a primer on public health measures that can be found in the Supplementary Materials. We then randomly assigned respondents into two conditions. One group received the following information on the projected economic costs of public health measures, while the other did not:

“But these actions will come at a cost. Economists project a recession and for as many as 660,000 Canadians to be out of work by the end of the year. The economy will not recover until social distancing is relaxed.”

Following the treatment, we asked our respondents how likely it is that they would do the following actions for an additional two months even if they are not sick: 1) avoid public gatherings; 2) avoid in-person contact; 3) avoid restaurants, bars, and shops; 4) avoid domestic and international travel. These measures were scaled from not at all likely (0) to extremely likely (3). We construct an index from these items (Cronbach’s alpha = .85). We measure expectations of adherence by other citizens using the same question battery but asking instead about “ordinary Canadians.” We create an index from these measures (Cronbach’s alpha = .84). Our outcome measures are standardized. The design of this experiment was informed by a pilot study we conducted on a sample of 2,495 adult English Canadians fielded from March 25–30, 2020. The pilot design and results can be found in the Supplementary Materials in S1 File.

We also examine whether treatment effects for our primary outcome vary by age. We present two models. One where we treat age as linear and continuous, and another where we construct a categorical measure – 18–34, 35–54, and 55 and older – to detect potential non-linearity in treatment effects. We control for possible confounders to heterogeneous effects in an identical fashion to the UESD study. Our results hold regardless of whether these controls are included (see Supplementary Materials). Descriptions of our variables and model estimates can be found in S2 Table in the Supplementary Materials in S1 File.

### Experiment 2

Our first experiment shows that exposure to negative economic information can reduce expectations of adherence with public health recommendations by others and by oneself. We believe that some of this latter effect is mediated by the former. We are only able to provide suggestive, non-causal evidence of mediation in this experiment because we were unable to exogenously manipulate expectations of other people’s public health adherence in the experiment (more details in the Supplementary Materials in S1 File).

We conducted a second experiment to provide direct evidence of a causal arrow running from expectations of public health adherence by other citizens and expectations of their own behavior. We surveyed 2,509 Canadian citizens 18 years or older between May 8–12, 2020 with sample provided by Dynata. Quotas were again set by Canadian region, language, age and gender, while weights are again applied within region by gender and age.

We randomly assigned respondents to receive polling information on the public’s willingness to engage in various public health precautions for an extended period of time to examine its effect on expectations of their own adherence. Respondents in treatment received information on the public’s willingness to comply with four different types of precautionary behavior. Those in control received none of this information.

We populate the data in our treatment with results from a similar survey of 2,504 Canadians conducted from May 1–5, 2020 where we asked respondents how many months they would be willing to: 1) avoid public gatherings; 2) avoid in-person contact; 3) avoid restaurants, bars, and shops; and 4) avoid domestic and international travel. We asked the same about their expectations of “ordinary Canadians” (categories: less than one month, one month, two months, three months, four months, five months, six months, more than six months).

**Fig 3:**
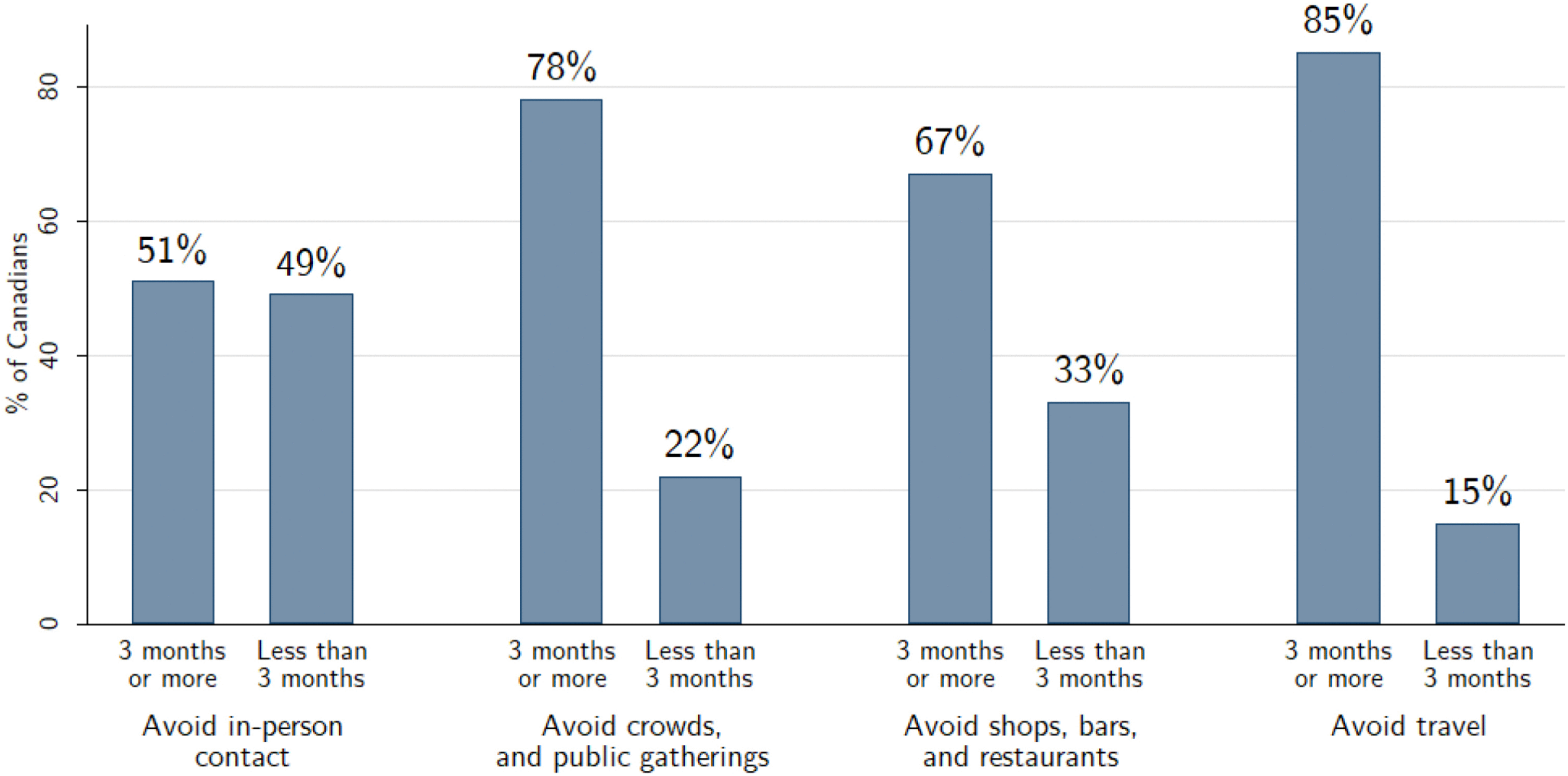
Treatment graph, Experiment 2.

Respondents underestimated the willingness of others to comply with public health recommendations in the long-term. 85% of respondents reported willingness to avoid travel for three or more months, but only 71% expected ordinary Canadians to do the same. 78% reported that they would avoid public gatherings for the same period of time, but only 55% expected others to do the same, while 67% would avoid shops, bars, and restaurants, with only 43% expecting the same of others. Respondents were divided on their willingness to avoid in-person contact for three months or more, with only 51% signaling such an inclination. We exposed our survey treated respondents to a graph illustrating these results, shown in Fig 3.

After the treatment we asked our respondents how many months they would be willing to: 1) avoid public gatherings; 2) avoid restaurants, bars, and shops; 3) avoid domestic and international travel; and 4) avoid in-person contact (categories: less than one month, one month, two months, three months, four months, five months, six months, more than six months). The first three items were used to construct a scale for our primary outcome (Cronbach’s alpha = .83), while the in-person contact item was used to as a placebo case where the polling information is conveying considerable uncertainty as to whether the public would maintain their adherence. As a manipulation check, we asked the same of their expectations of “ordinary Canadians” and constructed similar measures (Cronbach’s alpha = .82). We standardize each our outcome measures.

## Results – Survey experiments

### Experiment 1

Exposure to negative economic forecasts appears to affect expectations of adherence by other citizens and by themselves as expected. Respondents in the treatment condition scored 0.09 standard deviations lower in their expectations of others’ adherence compared to those given no information (p = .033). Respondents in the treatment condition reported expectations of their own adherence 0.10 standard deviations lower than those in the control (p = .011). These effects were mostly similar across subcomponents of the index. It was strongest for avoiding public gatherings (−0.11, p = .009) and avoiding restaurants, bars, and shops (−0.10, p = .011) and weakest and non-significant for avoiding domestic and international travel (−0.04, p = .265), with avoiding in-person contact closer in strength to the former than the latter (−0.09, p = .029). In short, information about the economic cost of public health recommendations appears to lower expectations of adherence by other citizens and by oneself, in support of H3 and H1B.

**Fig 4:**
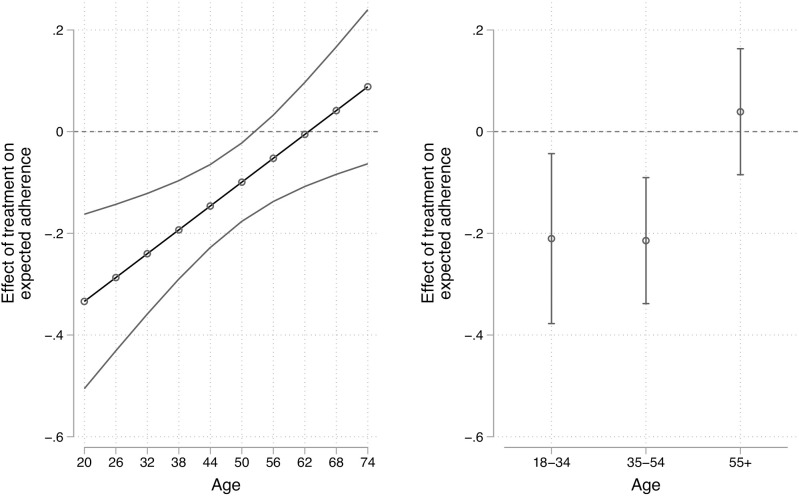
Heterogeneous effects of treatment on self-expectations of adherence across continuous (left) and categorical age measured (right). Note: 95% confidence intervals, controlling for treatment effects across levels of anti-intellectualism, left-right ideology, news exposure, education, and urban/rural residence. The full estimates can be found in S5 Table in S1 File.

The left panel of Fig 4 plots the estimated treatment effects across age controlling for potential confounders. Our prospective economic cost treatment is expected to lower expectations of public health adherence by 0.29 standard deviations among 26 year-olds, while there is no detectable effect among those 56 and older. The interaction term is statistically significant (p = .004). There does appear to be important non-linearity in these effects. Using the categorical measure of age, we observe nearly identical treatment effects for those aged 18−34 (−0.21, p = .014) and those aged 35−54 (−0.21, p = .001). There is no evidence of an effect among those older than the age of 55 (0.04, p = .536). In short, those who stand to benefit the most from public health measures are unresponsive to information about their costs, in support of H2B, and strikingly similar to our observational UESD results.

Again, we see some evidence that the effect of the economic information was strongest among those who thought their job was at least at some risk due to the pandemic (−0.18, p = .002) compared to those who believed there was no risk to their job (−0.03, p = .588), though the interaction term isn’t quite significant at conventional levels (p = .060). The point estimates are remarkably similar to the UESD results. These results are displayed in S4 Fig in the Supplementary Materials in S1 File.

### Experiment 2

The polling information we provided increased the length of time respondents believed others would engage in public health adherence. Exposure to the polling information is associated with a 0.21 standard deviation increase in expectations of public health adherence by other citizens (p < .001). This was not true for avoiding in-person contact – where polling indicated the public was divided and future adherence was much more uncertain (0.04, p = .309).

Exposure to the polling information is also associated with a 0.13 standard deviation increase in expected personal adherence (p = .002), in support of H4. Again, this was not true for the avoiding in-person contact item. Respondents were no more willing to avoid in-person contact when given information that the public was divided on this question (0.04, p = .290). We use a path model to estimate the share of the treatment effect that is mediated by changes in respondents’ expectations of adherence by other citizens. Close to 90% of the effect travels indirectly through changes in expectations of other people’s adherence, as we would expect. The direct effect is not statistically significant (p = .691).

We might be concerned about demand effects with an experiment such as this. People may infer the motivation of the researchers based on the presented data and adjust their responses accordingly. There are reasons to be skeptical of the importance of demand effects in survey experimental work [see 45], but a few other observations are inconsistent with demand effects driving our results. First, the strength of the effects we observe do not match the patterns displayed in the chart. Respondents saw the willingness of other citizens to avoid travel was particularly high, and yet effects were weaker on this dimension (0.08 SD, p = .050) than avoidance of public gatherings (0.14 SD, p < .001) and bars, restaurants, and shops (0.10, p = .010). Second. We do not observe any significant treatment effects on other similar variables measured immediately post-treatment, like precautionary behaviours taken over the past week (p = .633) or support for lockdown polices, like business and border closures, bans on travel, and fines for failing to physically distance (p = .317). Finally, we do not observe stronger treatment effects among people who might be motivated to respond to experimenter demand, like those with high COVID-19 risk perceptions and those who are highly trusting of experts. Neither interaction is substantively nor statistically significant (p = .679 & p = .758)

## Discussion

The COVID-19 pandemic imposed catastrophic economic costs on citizens as they – in part voluntarily – gave up personal liberties and refrained from social and economic activity to limit the spread of the novel coronavirus and protect others, as countries raced toward developing a vaccine and effective, scalable testing and tracing mechanisms. We need to understand the degree to which such behavior is sustainable, and, if it is not, identify policy solutions to ameliorate non-adherence.

We provide evidence that voluntary public health adherence is undermined by its economic consequences. We show that self-reported precautionary behavior declined after the release of the catastrophic jobs report on May 8, 2020, while aggregate-level mobility rose (H1A). We find this result to be the strongest among the young (H2A). We provide even more direct evidence of a causal relationship through a survey experiment. Exposing people to information about the economic costs of public health adherence reduces expectations of one’s own adherence in the future (H1B), particularly among the young (H1B), echoing our observational findings.

There are several mechanisms that can explain how economic shocks could undermine public health adherence. We argue that some of the effect results from changes in expectations of the behaviors of others in society. Exposing people to information about the economic costs of public health adherence reduces expectations of adherence by other citizens in the future (H3). We also provide causal evidence that expectations of public health adherence by others affect one’s own expected behavior (H4).

Although we can shed some light on our posited norm-based mechanism for how economic shocks reduce public health adherence, we cannot rule out other mechanisms and we fully expect others to be operating. It is quite likely that some people reduce their public health adherence because the economic information revealed the cost of precautionary behaviours to society and themselves irrespective of their expectations about others’ behaviour. Young people may have been particularly vulnerable to this dynamic because they bore the brunt of the costs for pandemic restrictions and received comparatively less benefit, owing to their greater resilience in the face of infection. Indeed, we see some evidence that the effects of the jobs report or information about economic costs are stronger among those at risk of COVID-19 job loss (S4 and S7 Figs in S1 File). Our observational data doesn’t allow us to test our norm-based mechanism, so it is possible that the effect we observe is some combination of direct effects of the economic information on adherence, and indirect effects through changes in beliefs about norms around COVID-19.

Likewise, there may be other reasons why young people are more responsive to the economic information in both the observational and experimental settings, though some of these expectations push in the opposite direction of our findings. Young people tend to have a higher tolerance for risk [46], but that would lead us to expect older people to be most responsive to information that conveys the economic consequences of pandemic policy. Younger people are also less trusting of official sources of information [47]. This cuts both ways, on the one hand potentially leaving them less trusting of, and less exposed to, official sources of information about the economic costs of pandemic policy, while on the other hand increasing their vulnerability to factors that might discourage adherence to state-endorsed behaviours. We control for trust in experts in our UESD and experimental analyses to block that mechanism, but we cannot rule it out entirely. The reality is that young people believed very different things about the pandemic than other citizens and often acted accordingly. We do not have a full account of why this is the case. It deserves more research.

Our analysis has some other limitations. First, our experiments hinge on the reliability of measuring people’s self-reported behavior (and their expectations of future behavior in the case of the experiments). We do, however, expect such error to be largely orthogonal to our experimental treatments and not a serious problem for the inferences we make here. Moreover, some recent work suggests that physical distancing self-reports do a good job at predicting people’s real world behavior [43]. Our analyses related to the April jobs report that use both self-reported behavior and behavioral data helpfully supplement our experiments that focus on behavioral intentions.

Second, while we are confident that the April jobs report produced substantial negative economic information, we cannot completely rule out the possibility that some other event occurred at the same time to produce a decline in public health adherence. That being said: 1) we find equivalent effects when narrowing the bandwidth of our analysis to closer proximity to the release date; 2) we still find effects controlling for COVID caseload and temperature changes in our aggregate-level analysis; and 3) we consistently find null effects for our placebo and falsification tests.

Our findings highlight the critical importance of research maintaining societal adherence to public health guidelines. The COVID-19 pandemic placed enormous social and economic burdens on citizens. More research is needed on how citizens adapt to these circumstances and how government policy can ensure such adaptation is socially optimal. Such research can help us understand not only how public health adherence can be maintained, but how social dilemmas more generally can be solved.

## Supporting information

S1 File Supplementary materials can be found in Supplementary Materials.docx. Replication data and code can be found at the following link: https://osf.io/q64uv/.
(DOCX)
