# Supplementary material for: Economic shock and the erosion of COVID-19 precautionary behavior in Canada during the early pandemic
Source: PLoS One. 2026 Feb 5;21(2):e0340685. doi: 10.1371/journal.pone.0340685 (PMC12875510; doi:10.1371/journal.pone.0340685)
Supplement: S1 File — Replication data and code can be found at the following link: https://osf.io/q64uv/. (DOCX) [file pone.0340685.s001.docx]

# **Supplementary Materials**

*Economic Shock and the Erosion of COVID-19 Precautionary Behavior in Canada During the Early Pandemic*

**Contents**

[Sample Characteristics 1](#_Toc210646240)

[Variable Descriptions 1](#_Toc210646241)

[COVID-19 Precaution Primers 2](#_Toc210646242)

[Pilot Experiment 3](#_Toc210646243)

[Mediation Analysis – Experiment 1 5](#_Toc210646244)

[Heterogeneous Effects Analysis – Experiment 1 9](#_Toc210646245)

[Economic News Sentiment Analysis 11](#_Toc210646246)

[Survey Fielding Dates and Attrition 13](#_Toc210646247)

[Treatment Compliance – Unexpected Event during Survey Design 14](#_Toc210646248)

[Balance Tests – Unexpected Event during Survey Design 15](#_Toc210646249)

[Heterogeneous Effects – Unexpected Event during Survey Design 16](#_Toc210646250)

[National and Provincial Mobility Series 18](#_Toc210646251)

# **Sample Characteristics**

**Table S1.** Sample characteristics

| Experiment = |  | Pilot | 1 | 2 |
| --- | --- | --- | --- | --- |
|  |  | March 25-30 | April 2-6 | May 8-12 |
| Female |  | 51.5 | 51.1 | 51.5 |
| Age | 18-34 | 27.3 | 27.2 | 25.2 |
|  | 35-54 | 34.2 | 33.5 | 34.2 |
|  | 55+ | 38.6 | 39.3 | 40.5 |
| University educated |  | 40.0 | 36.2 | 38.8 |
| French |  | 0.0 | 20.4 | 20.3 |
| Region | Atlantic | 7.9 | 6.9 | 6.9 |
|  | Quebec | 10.3 | 22.9 | 22.7 |
|  | Ontario | 45.1 | 38.3 | 38.6 |
|  | West | 36.8 | 31.9 | 31.7 |
| 2019 Liberal vote |  | 39.4 | 35.8 | 37.1 |
| N |  | 2495 | 2499 | 2509 |

# **Variable Descriptions**

**Table S2.** Variable descriptions

| **Measure** | **Description** |
| --- | --- |
| Public health precautions (UESD-only) | 0-1; index of three behaviors over the past week: 1) Avoided in-person contact with friends, family, and acquaintances; 2) Maintained 2 meters of distance from people as much as possible; 3) Avoided domestic travel. |
| COVID-19 risk perceptions | 0-1; How serious of a threat do you think the coronavirus is to Canadians? (very, somewhat, not very, not at all) |
| Anti-intellectualism | 0-1; Below is a list of groups and institutions in society. Please tell us the degree to which you trust or distrust members of these groups or institutions: 1) Experts; 2) Economists; 3) Scientists; 4) Doctors and medical professionals; 5) University professors (distrust a lot, distrust somewhat, neither, trust somewhat, trust a lot, don't know) |
| Ideology | 0-1; 1) The government should take measures to reduce differences in income levels; 2) Protecting the environment is more important than creating jobs; 3) Canada should increase the number of immigrants it admits each year; 4) People who don't get ahead should blame themselves, not the system; 5) The government should see to it that everyone has a decent standard of living (Strongly, somewhat, neither agree/disagree). Each item coded in left-wing (-1) and right-wing (1) direction. Don't knows and neither coded as neutral (0) |
| News exposure | Logged sum of exposure to following outlets in past week: 1) CBC; 2) CTV; 3) Global; 4) CityNews; 5) Globe and Mail; 6) National Post; 7) Toronto Star; 8) Local newspaper; 9) TVA (French-only); 10) TV5 (French-only); 11) La Presse (French-only); 12) Journal de Montreal (French-only); 13) Journal de Quebec (French-only); 14) Le Devoir (French-only); 15) Radio-Canada (French-only); 16) Rebel Media; 17) National Observer; 18) Toronto Sun; 19) The Tyee; 20) Post Millennial; 21) APTN; 22) True North News; 23) Press Progress; 24) Huffington Post; 25) Other |
| Education | Highest level of education: no schooling; some elementary school; completed elementary school; some secondary/high school; completed secondary/high school; Some technical, community college, CEGEP, College Classique; Completed technical, community college, CEGEP, College Classique; Some university; bachelor’s degree; master’s degree; professional degree or doctorate; don’t know |
| Age | Age in years |
| Urban/rural | Thinking about the place where you live, what word best describes it: A large city, a medium sized city, a large town, a small town, a rural place. |
| Gender | 1= female |
| Region | Province of residence: Atlantic = Newfoundland and Labrador, Prince Edward Island, Nova Scotia, New Brunswick; Quebec; Ontario; West = Manitoba, Saskatchewan, Alberta, British Columbia |

# **COVID-19 Precaution Primers**

Pilot Experiment

The coronavirus or COVID-19 is highly contagious. A person who carries the virus spreads it to an average of 3 people.

In an effort to control the spread of the virus, public health officials have called on Canadians to engage in social distancing where they limit in-person contact with high risk individuals, avoid common greetings, such as handshakes, steer clear of crowded places and non-essential gatherings, and keep a distance of two meters from others as much as possible.

Experts believe that these efforts can protect Canadians by limiting the coronavirus’ strain on the health care system.

Experiment 1

In an effort to control the spread of the coronavirus, public health officials have called on Canadians to engage in social distancing where they keep a distance of two meters from others as much as possible, limit in-person contact with high risk individuals, avoid travel, and steer clear of crowded places like bars, shops, restaurants, and non-essential gatherings. This is best achieved by staying at home as much as possible.

# **Pilot Experiment**

We conducted our pilot experiment on an online sample of 2,495 Canadian citizens 18 years or older collected from March 25-30, 2020 by Dynata. Quotas were set by Canadian region (Atlantic, Quebec, Ontario, and West), age and gender, while weights are applied within region by gender and age as well for the following analyses. The survey for the first experiment was conducted in English only, and as a result Quebec is underrepresented in this sample. The demographic breakdowns for all three surveys can be found above. The purpose of this experiment was to identify any threshold of job or unemployment loss with which we might observe effects on expectations of public health compliance and whether this information was more effectively conveyed through the unemployment rate or job numbers.

Design

We exposed all of our respondents to a primer on the precautions called for by public health officials found above. Immediately following its passage, respondents were randomly assigned information about the economic consequences of these actions. One group of respondents was told the economic consequences were uncertain:

Economists, to date, are uncertain of the economic consequences of these changes to Canadian life.

Other respondents were informed of possible economic consequences, with groups receiving either information on job losses:

These changes to Canadian life are expected to have economic consequences. Economists forecast a loss of [165/330/495/660],000 jobs by the third quarter of 2020.

Or the unemployment rate:

Economists forecast an increase in the unemployment rate to [6.7/7.7/8.7/9.7]% by the third quarter of 2020.

We randomly assigned both the scale of projected economic costs and the manner in which that information is communicated because we were uncertain as to the dosage required for a treatment effect and the most effective manner of conveying this information. We have strong priors that treatment effects will be higher as projected costs ratchet upwards and weak priors that people will be more responsive to job loss information rather than the unemployment rate, since some research has shown that people have little grasp of unemployment rates (Ansolabehere et al., 2011; Bullock et al., 2015).

Following the experiment, we asked our respondents how likely it is that “ordinary Canadians” would do the following actions for an additional two weeks even if they are not sick: 1) avoid public gatherings; 2) avoid in-person contact; 3) avoid restaurants, bars, and shops. These measures were scaled from not at all likely (0) to extremely likely (3). We construct an index from these items ranging from 0-1 (Cronbach’s alpha = 0.87). We standardize our outcome measure. We expect that respondents exposed to higher levels of projected economic cost will have lower expectations of social distancing compliance by other citizens.

**Figure S1.** Effects of prospective economic cost treatment on expectations of public health compliance by ordinary Canadians. Note: 95% confidence intervals.

Results

We find some evidence that information on economic cost lowers the expectations of respondents that others will take COVID-19 precautions. Individuals who were provided *any* economic information had expectations of compliance 0.08 standard deviations lower than the control condition, though this is not significant (p = 0.109). This effect is much stronger in the job loss conditions (-0.13, p = 0.017) than in the unemployment rate conditions (-0.03, p = 0.606).

We plot the effects of all our treatment conditions independently on expectations of public health compliance by other citizens in Figure S1. Our respondents appear to be comparatively more responsive to job losses. Information about a predicted loss of 165,000 jobs lowers expectations of the social distancing compliance by 0.14 standard deviations, though this is only marginally significant (p = 0.064). 495,000 jobs reduces the outcome by 0.15 standard deviations, which is also marginally significant (p = 0.062), while 660,000 jobs significantly reduces it by 0.17 standard deviations (p = 0.034). The corresponding effects for unemployment rate information are non-significant, though they are signed in the correct direction. The full estimates can be found in Table S3.

**Table S3.** Pilot study estimates

|  | Unemployment Rate | | Job Losses | |
| --- | --- | --- | --- | --- |
|  | Coef. | SE | Coef. | SE |
| 165,000/6.7% | 0.02 | 0.07 | -0.14* | 0.08 |
| 330,000/7.7% | -0.01 | 0.08 | -0.06 | 0.08 |
| 495,000/8.7% | -0.02 | 0.08 | -0.15* | 0.08 |
| 660,000/9.7% | -0.11 | 0.08 | -0.17** | 0.08 |
| Constant | 0.07 | 0.05 | 0.07 | 0.05 |
| N | 1476 | | 1494 | |

* p<0.1, ** p<0.05, *** p<0.01

# **Mediation Analysis – Experiment 1**

The results of Experiment 1 show that information regarding the high prospective economic cost of public health recommendations lowers both expectations of compliance by other citizens and expectations of their own compliance. We expect that at least some of the treatment’s effect on self-expectations of compliance is mediated by changes in expectations of other people’s behaviour. We attempted to test this theory by using a 2x2x2 between subjects, manipulation of the mediator design in Experiment 1.

In addition to assigning information about economic cost, we also randomly assign information signaling the public’s support for social distancing using data from our surveys:

Upwards of 85% of Canadians signal continued willingness to engage in social distancing practices according to public opinion polls.

Our expectation is that the negative effect of economic cost will be weaker among respondents assigned to this condition since we have set the mediator at a high level.

Mediation analysis poses extraordinary challenges. Experimentally manipulating the mediator is not a panacea because its manipulation could activate other traits or considerations outside of your causal logic that influences the outcome (Bullock et al., 2010). In this case, we have reason to believe that high prospective economic costs and lowered expectations of compliance by other citizens could create an expectation that governments will fail to continue to enforce public health guidelines. This, in turn, could influence prospective compliance for reasons that have nothing to do with the failure of others to engage in precautionary behaviour. Consequently, we randomly assign information that federal and provincial government intend to stand by public health guidelines as long as public health officials advise, while those in the control condition received no such information:

Federal and provincial governments intend to stand by social distancing guidelines as long as public health officials deem them necessary, which may be for weeks, if not months.

Our expectation is that the effect of economic cost will be weaker among respondents given this information. It should also directly affect expectations of one’s own social distancing.


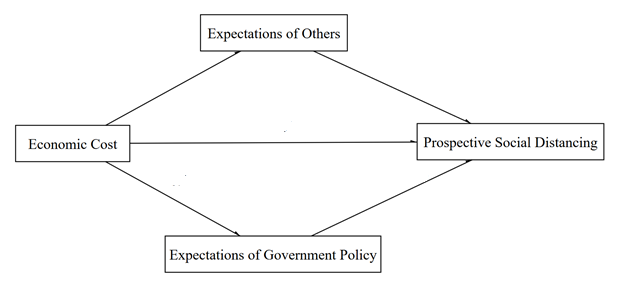


**Figure S2.** Path model

Method

We take two approaches to studying mediation in Experiment 1. First, we estimate the path model shown in Figure S2 that specifies a mediating effect of expectations on self-expectations of social distancing compliance. Path models allow for the testing of directional effects specified by a particular theory. In this case, it will allow us to estimate the direct effect of our exogenous economic cost treatment on self-expectations of public health compliance and the indirect effect that flows through changes in expectations of other citizens’ behavior and of government policy.

This analysis, however, is intended only to be descriptive. Path models require strong assumptions about causal direction and an absence of confounding that is nearly impossible to meet in social science (Green et al. 2010). We cannot rule out the existence of unmeasured mediators acting as confounds by being correlated with our mediators and the outcome. Nor can we be sure that the causal direction is from expectations of compliance by other citizens to self-expectations of compliance rather than the other way around. In short, we have causal identification between our economic cost treatment and our mediators and between our treatment and our outcome, but not between our mediators and the outcome.

As a result, we randomly assigned information to respondents to manipulate our mediating variables. We estimate the following models interacting each mediator treatment with economic cost. Our expectation is that treatment effects would be weaker when providing information that sets the mediator at a high level for respondents. A positive and significant coefficient on β_3_ and a negative coefficient on β_1_ would provide support for a mediating relationship:

$$self-expectations= \alpha+ \beta_{1}cost+\beta_{2}public support+\beta_{3}cost*public support+\beta_{4}government support+\beta_{5}cost*government support+\varepsilon$$

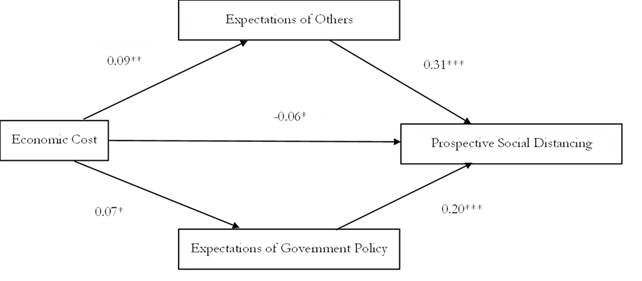


**Figure S3.** Direct effects of path model

Results

The direct effects of our path model are shown in Figure S3. The results indicate that about 60% of the total effect flows directly from economic cost to prospective social distancing (0.06 standard deviations, p = 0.09), while 40% flows indirectly through expectations of others and government policy (0.04, p = 0.02). That is, these results suggest that expectations of public and government compliance with social distancing partially mediate the effect of high prospective economic cost on self-expectations of social distancing.

This analysis, however, is descriptive. It does not provide causal identification between the mediating variables and the outcome. Unfortunately, our experimental results provide no evidence that treatment effects were weaker among respondents given information related to government policy or public support for social distancing, as is shown in model 1 of Table S4. In fact, the signs on the interaction terms are in the wrong direction.

This is likely a result of our treatments failing to manipulate our mediators of interest. Model 2 shows that information on public support for social distancing failed to increase expectations of social distancing compliance by ordinary citizens (p = 0.22). Model 3 illustrates that expectations of government policy are similarly not affected by information that government social distancing guidelines will continue indefinitely in response to advice of health experts (p = 0.63). Both the estimates are signed in the wrong direction. We can thus only say that our analyses provide suggestive evidence of mediation, but we do not have causal identification of the link between our mediator and the outcome. Therefore, we turn to Experiment 2 for evidence of a causal link between expectations of compliance by other citizens and self-expectations of compliance.

**Table S4.** Experimental mediation analysis and manipulation checks

|  | Expectations | | |
| --- | --- | --- | --- |
|  | Self | Others | Policy |
|  | 1 | 2 | 3 |
| Economic Cost Treatment | -0.02 |  |  |
|  | (0.07) |  |  |
| Government Support Treatment | 0.06 |  | -0.02 |
|  | (0.06) |  | (0.04) |
| Cost * Government Support Treatment | -0.11 |  |  |
|  | (0.08) |  |  |
| Public Support Treatment | -0.02 | -0.05 |  |
|  | (0.06) | (0.04) |  |
| Cost * Public Support Treatment | -0.05 |  |  |
|  | (0.08) |  |  |
| Constant | 0.03 | 0.02 | 0.01 |
| N | 2489 | 2489 | 2489 |

Standard errors in parentheses; * p<0.1, ** p<0.05, *** p<0.01

References

Green, Donald P., Shang E. Ha, and John G. Bullock. 2010. “Enough Already About ‘Black Box’ Experiments: Studying Mediation Is More Difficult Than Most Scholars Suppose.” *The Annals of the American Academy of Political and Social Science* 628 (1): 200–208. <https://doi.org/10.1177/0002716209351526>.

# **Heterogeneous Effects Analysis – Experiment 1**

**Table S5.** Heterogeneous effects estimates, Experiment 1

|  | Continuous | | | | | | | | Categorical | | | | | | | |
| --- | --- | --- | --- | --- | --- | --- | --- | --- | --- | --- | --- | --- | --- | --- | --- | --- |
|  | Coef. | | SE | | Coef. | SE | | Coef. | | | SE | Coef. | | | SE |  |
| Treatment | -0.444 | | 0.137 | | -0.435* | 0.254 | | -0.179** | | | 0.086 | -0.167 | | | 0.229 |  |
| Age | 0.007*** | | 0.002 | | 0.007*** | 0.002 | |  | | |  |  | | |  |  |
| Treatment * Age | 0.007*** | | 0.003 | | 0.008*** | 0.003 | |  | | |  |  | | |  |  |
| 35-54 |  | |  | |  |  | | 0.321*** | | | 0.070 | 0.331*** | | | 0.069 |  |
| 55+ |  | |  | |  |  | | 0.312*** | | | 0.074 | 0.319*** | | | 0.075 |  |
| Treatment * 35-54 |  | |  | |  |  | | -0.027 | | | 0.107 | -0.004 | | | 0.106 |  |
| Treatment * 55+ |  | |  | |  |  | | 0.227** | | | 0.108 | 0.250** | | | 0.107 |  |
| Anti-intellectualism |  | |  | | -0.133*** | 0.038 | |  | | |  | -0.137*** | | | 0.039 |  |
| Treatment * Anti-intellectualism | |  | | -0.101* | | | 0.057 | |  |  | | | -0.098* | 0.058 | | |
| Ideology |  | |  | | -0.028** | 0.012 | |  | | |  | -0.027** | | | 0.012 |  |
| Treatment * Ideology |  | |  | | -0.002 | 0.019 | |  | | |  | 0.001 | | | 0.019 |  |
| News Exposure |  | |  | | 0.045 | 0.054 | |  | | |  | 0.06 | | | 0.054 |  |
| Treatment * News Exposure |  | |  | | 0.026 | 0.079 | |  | | |  | 0.016 | | | 0.079 |  |
| Education |  | |  | | 0.038** | 0.015 | |  | | |  | 0.037** | | | 0.015 |  |
| Treatment * Education |  | |  | | -0.017 | 0.022 | |  | | |  | -0.013 | | | 0.021 |  |
| Urban/Rural |  | |  | | -0.017 | 0.022 | |  | | |  | -0.017 | | | 0.022 |  |
| Treatment * Urban/Rural |  | |  | | 0.052 | 0.034 | |  | | |  | 0.051 | | | 0.034 |  |
| Constant |  | |  | | -0.463*** | 0.173 | |  | | |  | -0.357** | | | 0.158 |  |
| R^2^ | 0.038 | | | 0.078 | | | | |  | 0.035 | | | 0.077 | | | |
| N | 2489 | | | 2486 | | | | |  | 2489 | | | 2486 | | | |

* p<0.1, ** p<0.05, *** p<0.01

**Figure S4.** Heterogeneous effects of treatment across risk of job loss due to the COVID-19 pandemic. Note: 95% confidence intervals.

# **Economic News Sentiment Analysis**


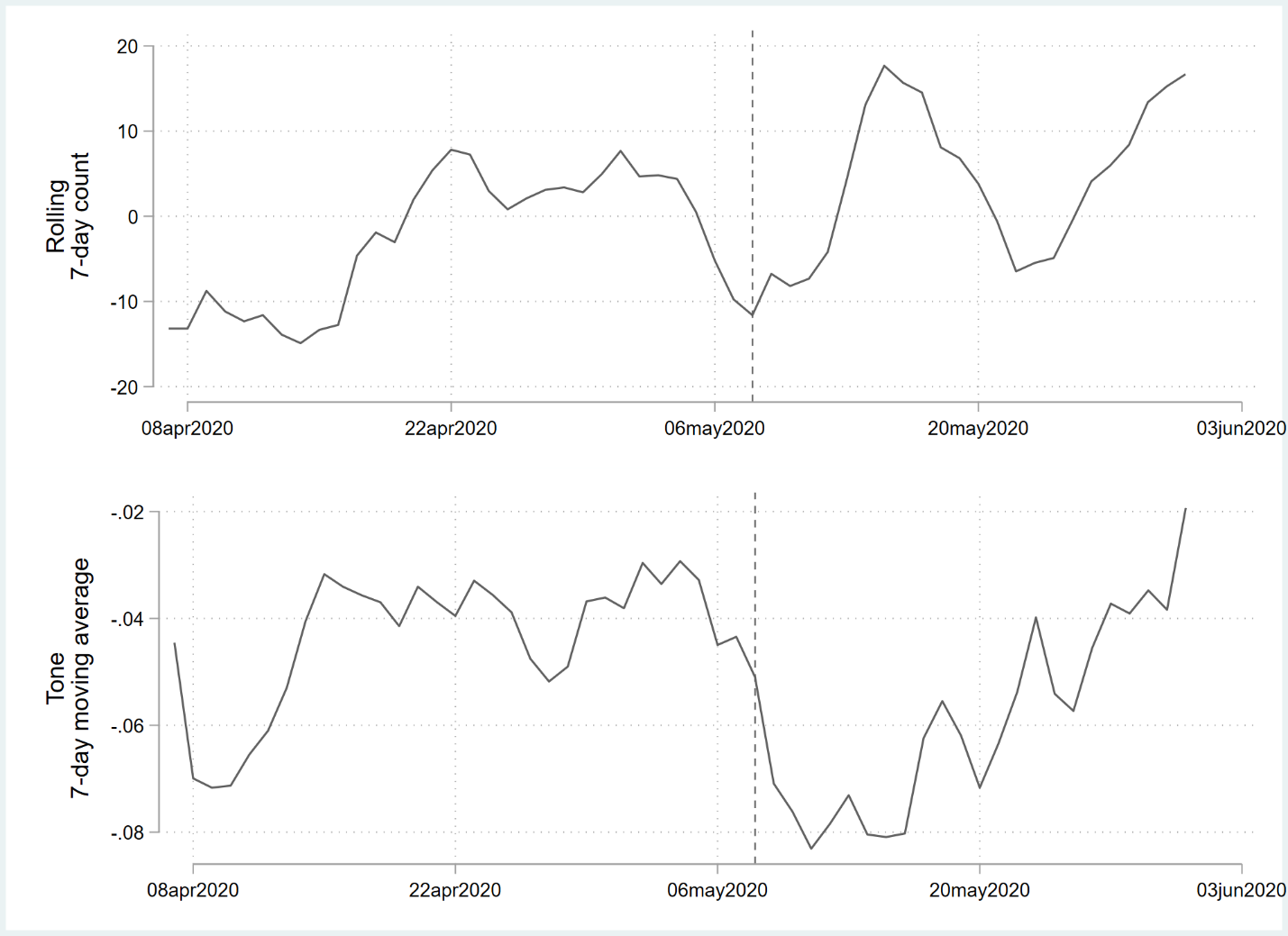


**Figure S5.** 7-day rolling sum of economic news content (top); 7-day moving average of headline sentiment (middle); composite coverage negativity index (bottom). Dashed line represents release of the April jobs report.

The April jobs report precipitated a torrent of negative news about the economy. We downloaded all newspaper headlines between April 1 and May 27 from Canada’s two national circulating newspapers from *Lexis Uni* – the *Globe and Mail*, and the *National Post*, along with two major regional papers, the *Toronto Star*, and the *Montreal Gazette* (N=5,422). Headlines were selected if they contained the following keywords in the body of the article: (econom* or jobs or employment) and Canada. These words were used to restrict our search to economic news focused on Canada. The top panel of Figure S4 contains a 7-day rolling sum of economic news articles from these four sources. The dashed line indicates the day the jobs report was released. A sharp spike in economic news coverage clear coincides with the release of the report.

We can also measure the sentiment of our headlines. We use the economic sentiment dictionary of Hopkins et al. (2017) that counts negative and positive economic terms to evaluate tone in economic news content.^[[1]](#footnote-2)^ We apply this dictionary to our headlines and calculate a tone score per article by taking the difference between the positive and negative words, which we average daily. The middle panel of Figure S4 displays a 7-day moving average of tone in our economic news headlines, where the dashed line represents the release of the jobs report. As is clear, the release of the report coincides with a sharp and sustained drop in headline sentiment. We replicate our main findings with the Lexicoder Sentiment Dictionary (Young and Soroka 2012) as well, which can be found in Figure S5. We take this as compelling evidence that the release of the economic jobs report provided an information shock about the economic costs of public health recommendations to the mass public.


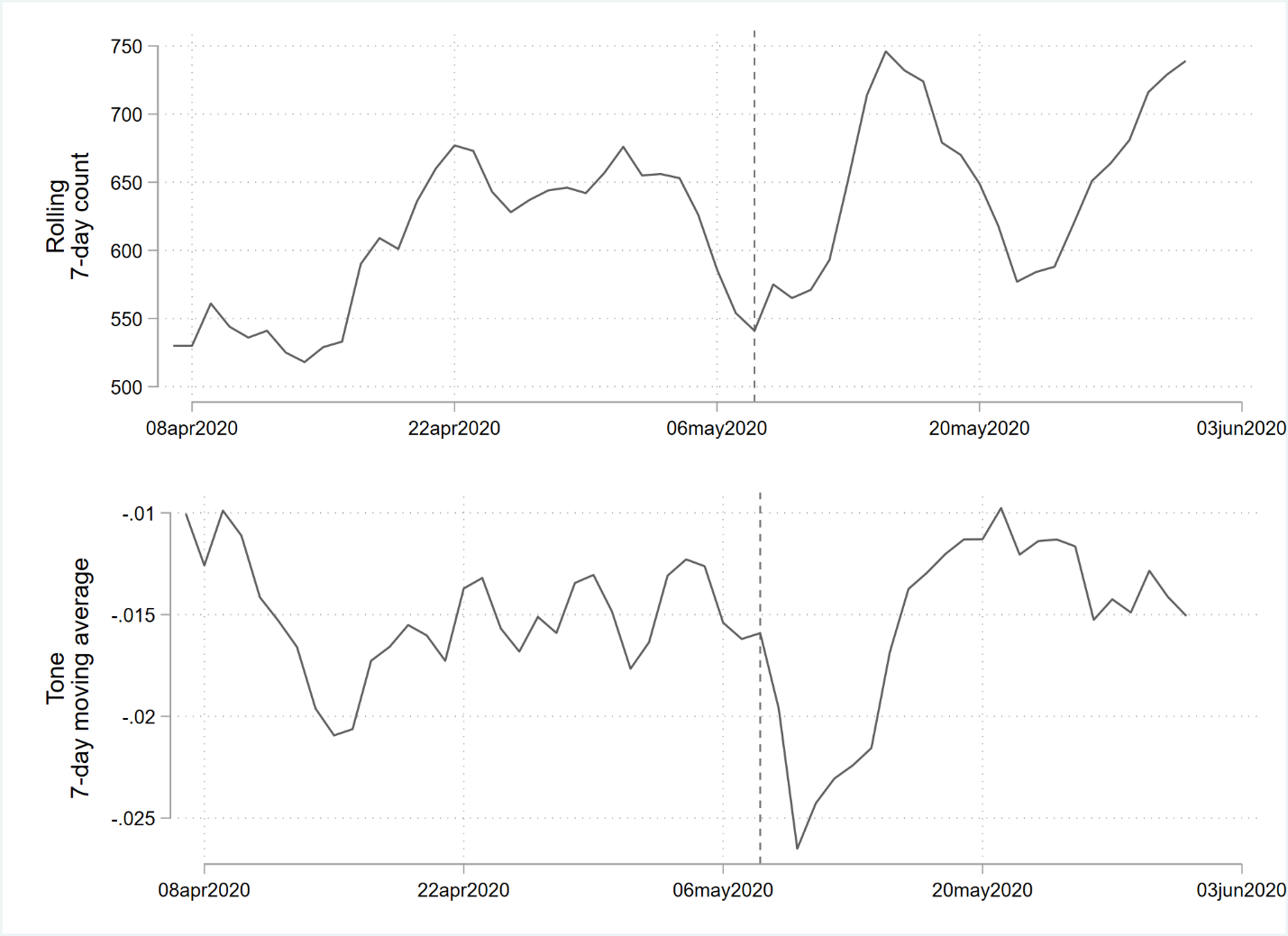


**Figure S6.** 7-day rolling sum of economic news content (top); 7-day moving average of headline sentiment (middle); composite coverage negativity index (bottom). Dashed line represents release of the April jobs report.

References

Hopkins, Daniel J., Eunji Kim, and Soojong Kim. 2017. “Does Newspaper Coverage Influence or Reflect Public Perceptions of the Economy?” *Research & Politics* 4 (4). <https://doi.org/10.1177/2053168017737900>.

Young, Lori, and Stuart Soroka. 2012. “Affective News: The Automated Coding of Sentiment in Political Texts.” *Political Communication* 29 (2): 205–31. <https://doi.org/10.1080/10584609.2012.671234>.

# **Survey Fielding Dates and Attrition**

**Table S6.** Survey waves and fielding dates

| Wave | Fielding Date | N | Recontacts |
| --- | --- | --- | --- |
| 1 | April 2-6, 2020 | 2,489 | 0 |
| 2 | April 9-11, 2020 | 2,493 | 0 |
| 3 | April 16-19, 2020 | 2,489 | 0 |
| 4 | April 24-29, 2020 | 2,515 | 900 |
| 5 | May 1-5, 2020 | 2,512 | 928 |
| 6 | May 8-12, 2020 | 2,514 | 962 |
| 7 | May 21-27, 2020 | 2,527 | 891 |

**Table S7.** Sample characteristics for initial contacts and successfully recontacted respondents

|  |  | Initial sample, Wave 1-3 | Recontacts, Wave 4-7 | Attrition |
| --- | --- | --- | --- | --- |
| Female |  | 51.2 | 49 | -2.2% |
| Age | 18-34 | 26.7 | 19.6 | -7.1% |
|  | 35-54 | 33.6 | 38.1 | +4.5% |
|  | 55+ | 39.7 | 42.3 | +2.6% |
| French |  | 20.7 | 25 | +4.3% |
| Region | Atlantic | 6.9 | 5.9 | -1.0% |
|  | Quebec | 23.2 | 26.7 | +3.5% |
|  | Ontario | 38.2 | 37.4 | -0.8% |
|  | West | 31.7 | 30 | -1.7% |
| Degree |  | 37.4 | 39.9 | +2.5% |
| Political interest |  | 6.2 | 6.3 | +0.1% |
| N |  | 7471 | 3681 |  |

# **Treatment Compliance – Unexpected Event during Survey Design**

We asked respondents to give their best guess on the unemployment rate in Canada (response options: 3.6%, 5.6%, 7.6%, 10.6%, don’t know)^[[2]](#footnote-3)^. We re-scale this variable to range from 3.6 to 10.6 with “don’t knows” coded as missing. We should observe changing perceptions of unemployment in Canada induced by the report. If we do not, not only is compliance low, but this might be a sign that the report did not provide people with novel information. It is possible that economists, journalists and commentators preempted the release of the report or that people learned this information indirectly through their own personal experience.

We regress within-respondent changes in perceptions of the unemployment rate on a dummy variable indicating the release date (i.e., May 8) adjusting for covariate imbalance by age. Respondents re-contacted before the release of the report increased their perception of the unemployment rate by 0.4 percentage points (p < .001). Respondents re-contacted after the release of the report increased their perception of the unemployment rate by an additional 1.1 points (p < .001), which is about 0.44 standard deviations. We can restrict the bandwidth around the release date to the last wave of contacts and the re-contacts in the waves immediately before and after the release date (*Narrow 2*). Respondents re-contacted before the release of the report increased their perception of unemployment by a non-significant 0.08 points (p = .623). In contrast, respondents who were contacted after the release of the report increased their perception of unemployment by a nearly identical 1.1 points (p<.001) or 0.49 standard deviations. There appears to be a substantial increase in negative evaluations of Canada’s unemployment situation after the release of the report.

Not all respondents were equally treated, however. We estimate the above model when interacting news exposure with our jobs report release dummy variable. Respondents with no reported political news exposure in the previous week increased their perceptions of the unemployment rate by 0.45 points, which is not statistically significant (p = .173). Individuals in the 95 percentile of news exposure, in contrast, increased their perceptions of unemployment by 1.54 points (p < .001). The following estimates can be more appropriately classified as average intent to treat effects, rather than average treatment effects.

# **Balance Tests – Unexpected Event during Survey Design**

**Table S8.** Balance tests for UESD

|  | Base | Narrow 1 | Narrow 2 | Placebo |
| --- | --- | --- | --- | --- |
| 35-54 | 0.022** | -0.029*** | -0.124*** | 0.042*** |
|  | (0.011) | (0.009) | (0.031) | (0.014) |
| 55+ | 0.037*** | -0.045*** | -0.166*** | 0.046*** |
|  | (0.011) | (0.009) | (0.030) | (0.014) |
| Education | 0.001 | -0.004** | -0.012** | 0.001 |
|  | (0.002) | (0.002) | (0.006) | (0.003) |
| Income | -0.003 | 0.001 | 0.001 | -0.001 |
|  | (0.003) | (0.002) | (0.007) | (0.004) |
| Urban/rural | -0.002 | -0.000 | 0.001 | -0.004 |
|  | (0.003) | (0.002) | (0.008) | (0.004) |
| Female | 0.004 | -0.008 | -0.030 | 0.008 |
|  | (0.008) | (0.006) | (0.021) | (0.011) |
| Quebec | 0.008 | 0.010 | -0.015 | 0.050** |
|  | (0.019) | (0.014) | (0.049) | (0.024) |
| Ontario | 0.003 | -0.011 | -0.033 | 0.003 |
|  | (0.018) | (0.013) | (0.049) | (0.024) |
| West | 0.004 | -0.010 | -0.039 | 0.003 |
|  | (0.019) | (0.014) | (0.050) | (0.024) |
| Constant | 0.135*** | 0.127*** | 0.496*** | 0.273*** |
| N | 7944 | 7132 | 1757 | 7944 |

* p<0.1, ** p<0.05, *** p<0.01

# **Heterogeneous Effects – Unexpected Event during Survey Design**

**Table S9.** Heterogeneous effects estimates, UESD

|  | Continuous | | | | Categorical | | | |
| --- | --- | --- | --- | --- | --- | --- | --- | --- |
|  | Coef. | SE | Coef. | SE | Coef. | SE | Coef. | SE |
| Jobs report | -0.167*** | 0.057 | -0.230** | 0.105 | -0.125*** | 0.038 | -0.183** | 0.091 |
| Age | 0.000 | 0.000 | 0.001 | 0.001 |  |  |  |  |
| Report * Age | 0.003*** | 0.001 | 0.003*** | 0.001 |  |  |  |  |
| 35-54 |  |  |  |  | -0.008 | 0.026 | -0.005 | 0.026 |
| 55+ |  |  |  |  | 0.004 | 0.024 | 0.011 | 0.025 |
| Report * 35-54 |  |  |  |  | 0.111** | 0.046 | 0.114** | 0.046 |
| Report * 55+ |  |  |  |  | 0.136*** | 0.042 | 0.135*** | 0.044 |
| Anti-intellectualism |  |  | 0.012 | 0.014 |  |  | 0.012 | 0.014 |
| Report * Anti-intellectualism |  |  | -0.001 | 0.023 |  |  | -0.002 | 0.023 |
| Ideology |  |  | 0.00 | 0.004 |  |  | 0.00 | 0.004 |
| Report * Ideology |  |  | -0.003 | 0.007 |  |  | -0.003 | 0.007 |
| News exposure |  |  | 0.030 | 0.055 |  |  | 0.032 | 0.055 |
| Report * News |  |  | 0.078 | 0.090 |  |  | 0.076 | 0.090 |
| Education |  |  | 0.002 | 0.005 |  |  | 0.002 | 0.005 |
| Report * Education |  |  | 0.001 | 0.009 |  |  | 0.00 | 0.009 |
| Urban/rural |  |  | 0.010 | 0.007 |  |  | 0.01 | 0.007 |
| Report * Urban/rural |  |  | 0.009 | 0.012 |  |  | 0.01 | 0.012 |
| Constant |  |  | -0.119** | 0.055 |  |  | -0.086* | 0.048 |
| R^2^ | 0.019 | | 0.029 | | 0.022 | | 0.031 | |
| N | 1890 | | 1890 | | 1890 | | 1890 | |

* p<0.1, ** p<0.05, *** p<0.01

**Figure S7.** Intent-to-Treat effects of the job report release on changes in public health adherence across risk of job loss due to COVID-19. Note: 95% confidence intervals.

# **National and Provincial Mobility Series**

**Figure S8.** Comparison of adjusted and raw mobility series for national (left) and provincial mobility data (right). Note that adjusted series is constructed from model residuals after predicting mobility with a series of binary variables or holidays (Good Friday, Easter Sunday, and Victoria Day) and day of the week.

1. The keywords are as followings: fall*, recess*, problem, unemploy*, slump*, slow*, drop*, fear, debt, bad, plung*, jobless, loss*, bear, layoff* (negative); profit*, bull, growth, growing, grow, inflat*, invest* (positive). [↑](#footnote-ref-2)
2. We omit the last wave because the response categories were changed to reflect the dramatic increase in the unemployment rate as announced in the jobs report. [↑](#footnote-ref-3)
